# Containing COVID-19 and the social costs on human rights in African countries

**DOI:** 10.1057/s41599-022-01357-4

**Published:** 2022-10-03

**Authors:** Lenore Manderson, Diego Chavarro, Blessings Kaunda-Khangamwa, Alexander Kagaha, Henry Zakumumpa

**Affiliations:** 1 School of Public Health, University of the Witwatersrand, Johannesburg, South Africa; 2 School of Social Sciences, Monash University, Melbourne, VIC Australia; 3 ARIN (Africa Research and Impact Network), Nairobi, Kenya; 4 Kamuzu University of Health Sciences, Blantyre, Malawi; 5 University of Gothenburg, Gothenburg, Sweden; 6 CARTA (Consortium for Advanced Research Training in Africa), Nairobi, Kenya; 7 Makerere University, Kampala, Uganda

**Keywords:** Social policy, Health humanities

## Abstract

Multiple social interventions were introduced to contain the COVID-19 pandemic across Africa, limiting social engagement, school and workplace attendance, and travel. In anticipation of negative economic consequences and social impact, many governments introduced cash transfers, social pensions, food aid, and utility and tax waivers. However, people living precariously and/or under conditions of structural vulnerability were often unable to access to this support. A rapid review was undertaken on COVID-19 and the effects of interventions on human rights in African countries, examining primary studies, editorial notes, opinion papers, and literature reviews, with focus on qualitative approaches and discussions. In examining the links between health, human rights and non-pharmaceutical interventions on vulnerable populations, the review identified that: (1) people who were vulnerable were excluded from or not adequately represented in policy responses to COVID-19; (2) the precarious socio-economic conditions of these populations were not adequately addressed by dominant policy responses; and (3) only partial support was offered to those whose relationship with the state was ambiguous or conditional, so compromising human rights. Interactions between health, human rights, and underlying social and economic conditions amplified poor health and impoverishment of those who were already vulnerable. The challenge is to find a balance between stopping the spread of COVID-19 and the protection of human rights; to implement population-specific responses to supplement uniform public health responses; and to address causes (structural vulnerability) rather than symptoms. There is a need to plan rather than react to pandemics, and to co-construct interventions with rather than delivering instructions to populations. These recommendations serve as instruments to be considered when designing new policies, to incorporate a human rights perspective in responses to current and future pandemics.

## Introduction

Diverse non-pharmaceutical interventions (NPI) were introduced as the COVID-19 pandemic began to take hold in African countries in order to contain viral transmission, minimise infection, and ensure the preparedness of hospitals and clinics to provide appropriate care. These interventions included the temporary closure of schools and workplace sites, with shifts to educating and working from home where feasible, the cancellation of public events, restrictions on the size of public and private gatherings (including religious congregations, weddings, and funerals), suspension of public transport and restrictions on travel, imposition of curfews, and contact tracing (Hong et al., 2021). While considered necessary and consistent with those introduced worldwide, these measures have had tremendous impacts on human lives, particularly people disadvantaged by virtue of their living conditions, poverty, and pre-existing discriminatory and exploitative practices (Manderson and Levine, 2020). Governments across Africa anticipated social, health and economic impacts of these intervention measures, and to mitigate these, introduced various forms of social assistance, social insurance, and labour market policies. These included supplements to social pensions, cash transfers, and utility, food aid and tax waivers (Gentilini et al., 2021). Scholars have expressed concern, however, that neither the NPIs nor social policies considered the links between health and human rights (Sekalala et al., 2020).

In this review, we examine the links between health, human rights, and NPIs on vulnerable populations, and offer guidance for more integrated and holistic policy responses to COVID-19 in Africa. We draw on the review work we conducted under the programme on COVID-19 Science Engagement to Influence Government Policy, an activity of the Alliance for Accelerating Excellence in Science in Africa (AESA) platform (Chavarro et al., 2021). Our initial concerns were the short- and long-term social impacts of COVID-19 in Africa, and the socio-economic, cultural and contextual factors impacting on adherence to COVID-19 public health initiatives (NPIs). Addressing these questions led us to consider whose lives were overlooked as the NPIs were implemented—who were rendered invisible, whose lives were ungrievable (Butler, 2006). Our review aimed to draw out the policy implications of research on pandemic measures of containment and mitigation of their negative impact; as we illustrate, the pandemic resulted in unintended consequences for vulnerable populations and their human rights. Below, we:

- Provide a framework of human rights, structural vulnerability, health, and their interconnections to analyse public health policy responses to the COVID-19 pandemic;
- Identify and reflect on the impact of COVID-19 NPIs on the health, human rights, structural vulnerability, and conditions of everyday life of vulnerable populations in Africa; and
- Consider the implications of the review findings for integrated and holistic interventions and policy responses to COVID-19 in Africa.

## Conceptual framework

The impact of the pandemic on vulnerable populations was not addressed in the main policy document that helped frame an integrated response in Africa to COVID-19 to prevent “severe illness and death” and minimise “social disruption and economic consequences” (African Union, 2020, p. 3). The document did not question inclusion and exclusion criteria in state responses, and focused on disruption to social and economic life rather than on protecting human rights. While little research has yet been conducted on the immediate social and economic impacts of the pandemic, even less has been conducted on the impact of these NPIs on the human rights of vulnerable people (Chiwona-Karltun et al., 2021; Egger et al., 2021). We aim to bring a broad framework of human rights, structural vulnerability, health, and their interconnections, to be addressed in policy making in relation to pandemics.

Hygiene, social isolation, and strict controls of social interaction are the most effective public measures to contain a novel pandemic and its unknown effects (World Health Organization, 2014), although evidence of the mix of measures is not conclusive (Shongwe, 2020). In general, the NPIs introduced during the COVID-19 pandemic drew on conventional understandings of disease transmission and public health interventions, and while they anticipated and responded to the needs and priorities of medical and health services, they did not allow a broad and holistic perspective that included social and economic constraints and local contingencies (Mann et al., 1994; WHO Commission on Social Determinants of Health, 2008). Health policies can impact the human rights of citizens either positively or negatively; conversely human rights policies affect health, creating an interdependent and bidirectional relationship between the two (Mann et al., 1994). Structural vulnerability shapes and is an outcome of variations of health and human rights (Manderson and Levine, 2020). A broader framework recognises that human rights and health are inextricably linked (Sekalala et al., 2020; World Health Organization, 2021).

Structural vulnerability can be defined as “a positionality that imposes physical, social, emotional and economic suffering on specific population groups and individuals in patterned ways” (Quesada et al., 2011, p. 339). The term vulnerability draws attention to how “disparities of class, culture, gender, sex and race impact on individuals, families and communities” (Team and Manderson, 2020), with these disparities and their impact magnified in the context of the pandemic. Drawing on the published literature to 30 June 2021, we identified a range of populations who were vulnerable for various reasons; these included women, children and youth, people living in multidimensional poverty, residents of institutions, refugees, and people living with disabilities. At an economic level, vulnerability is reflected in decreasing living standards and growing hunger among many people in African countries (Deaton, 2021; Egger et al., 2021). However, the impact of pandemic NPIs extends well beyond this. We include structural vulnerability, referring to the administrative and legal conditions, including in relation to residency and citizenship, that place certain populations at heightened risk “for adverse health outcomes through their interface with socio-economic, political and cultural/normative hierarchies” (Bourgois et al., 2017, p. 17).

The significance of structural vulnerability during the COVID-19 pandemic is well illustrated by Garimella and colleagues (2021) in their account of the circumstances of “waste pickers” in India. These men, women and children survive by collecting and selling recyclables, typically living on site, on garbage dumps, to readily access deliveries of waste as they come to hand. The population has poor access to water, sanitation, electricity and services, and was invisible in public health policies even before the COVID-19 outbreak. The communities were particularly disadvantaged by pandemic measures that restricted people’s income generation activities, and by policies that did not recognise them explicitly as vulnerable and maintained them in a state of “debility.” Specific measures to reduce the transmission of coronavirus magnified their difficulties, as they sought to generate an income, living in conditions of extreme poverty, poor housing, without access to hygiene and sanitation services, subject to discrimination and marginalisation. Such constraints on livelihood are likely replicated worldwide among other communities, including across Africa, where people live in informal settlements and often subsist on grants, sporadic contract work, waste-collecting and recycling, small trade and begging (Buckley, 2020; Auerbach and Thachil, 2021; Collantes, 2021).

**Fig. 1:**
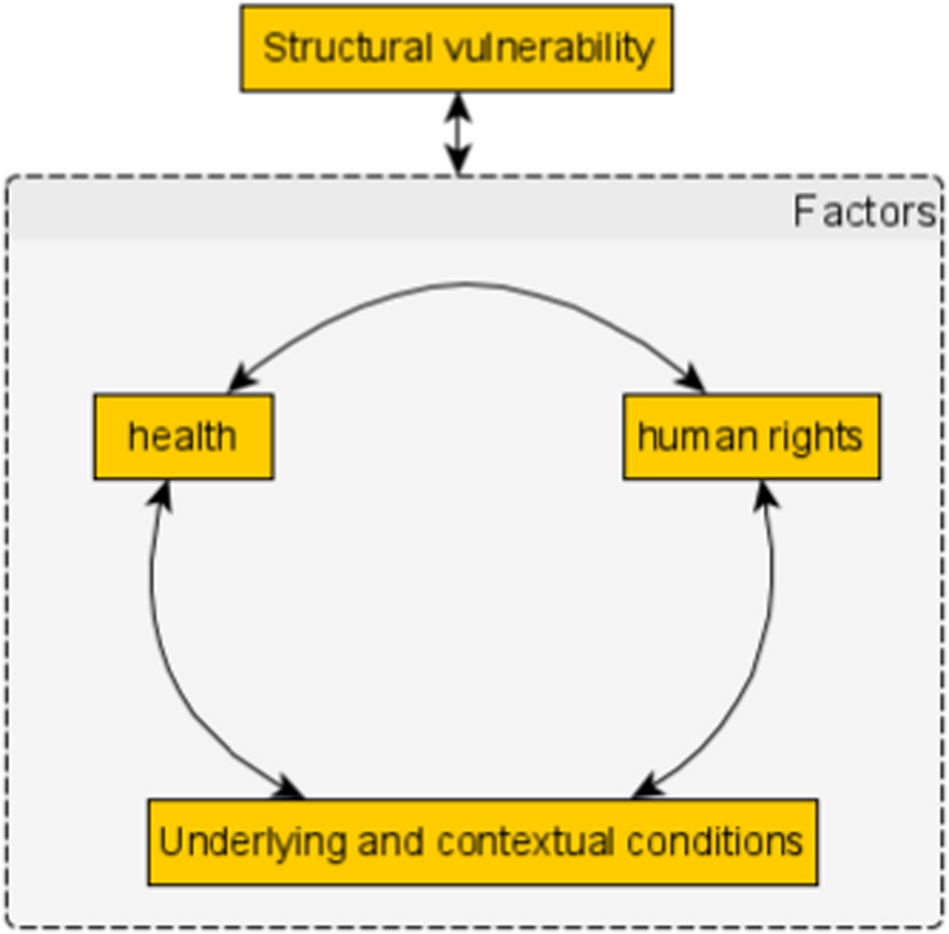
Initial conceptual framework. This shows the relationships between health, human rights, and underlying conditions as determinants of structural vulnerability.

In mapping a rapid review, and in our analysis of the links between health, vulnerability and human rights, we adopted an initial framework in which the multidirectional relationships between underlying conditions, health, and human rights both produced and were affected by structural vulnerability (Fig. 1).

The relationships in Fig. 1 imply that any policy intervention on health necessarily affects human rights, not only because the conditions for good health are a fundamental human right, but also because human rights are interdependent and non-hierarchical (Quane, 2012) By intervening on specific aspects of health, policymakers inevitably affect other aspects for consideration when designing such interventions.

## Methods

This article is based on work conducted under the auspices of the Africa Academy of Sciences (Chavarro et al., 2021). We focused on the peer-reviewed literature in which Africa was the main subject of analysis, whether at continental, national, or subnational level. The Scopus database produced the core dataset. We performed three different searches in this database. The first identified literature related to economic and structural vulnerability in Africa. The second search added a series of NPIs such as lockdowns and social distancing. The third included human rights. These searches were supplemented by Epistemonikos and the Cochrane Covid-19 register database of reviews. We focused our search on subject rather than on specific health-related disciplines, given that social, economic, environmental, and physical aspects were all relevant to the pandemic. Focusing on one discipline may have yielded more depth on a specific aspect, but would have given a limited perspective on human rights and policy making.

We included primary studies that used qualitative methods for data collection and analysis and studies that used secondary data, including quantitative data analysis. After performing the searches and deduplicating the records, two review authors (Manderson and Chavarro) screened titles and abstracts for studies meeting the key criterion of identifying connections between health, NPIs, vulnerable populations, and human rights. Women, children, migrants and refugees, and institutionalised residents, were the predominant groups identified as vulnerable; fewer studies focused on adolescents and young people, sex workers, sexual minorities, inmates, persons with medical co-morbidities, and homeless people. NPIs introduced to contain the pandemic included reduced social interaction through curfews, closures and limits to numbers of individuals, such as school and workplace closures, restricted numbers of people at public events (e.g., at religious services, weddings and funerals), changes in mobility (reduced public transport services, restricted private travel), and curfews, quarantine, and contact tracing (Hong et al., 2021). In this review, we define human rights as a domain rather than as an individual aspect of human relations, and refer to impact on human rights in relation to any actual or potential effect of an NPI on the rights of a population (Mann et al., 1994). We did not list all human rights to select the studies, but rather relied on the attention given to this by authors.

**Fig. 2:**
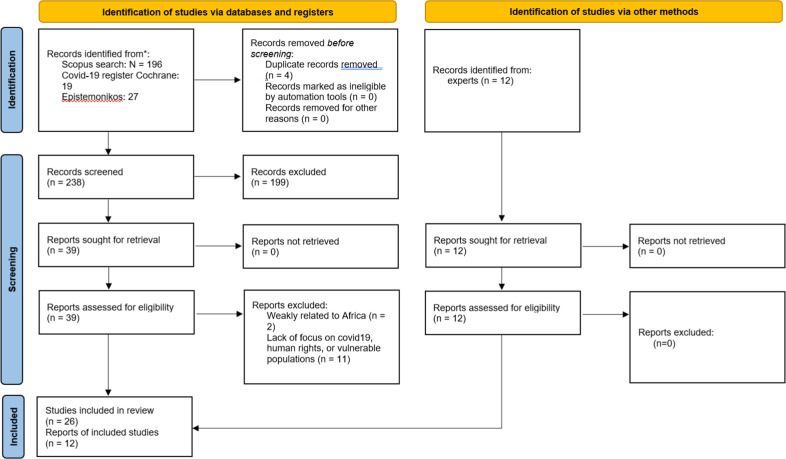
PRISMA flow diagram. This depicts our literature review search and selection procedure.

All potentially relevant papers were downloaded, and we selected those meeting the selection criteria based on full-text screening. We crosschecked these documents against our own expert knowledge to ensure we had identified as many relevant studies as possible. The final sample comprised 38 papers distributed as follows: fifteen commentaries, eleven law articles, five other qualitative and quantitative articles, four letters to the editor, two editorials, one case study and one case report. In terms of countries, the literature predominantly reported South Africa’s experience (*n* = 13), then Nigeria (*n* = 2), with one publication each for Malawi, Lesotho, Kenya, Uganda, Central African Republic, and South Sudan. Of those with a regional focus, 12 articles were concerned with Sub-Saharan Africa as a whole, two with Southern and East Africa, and two Western Africa. Two documents did not address a specific region. This uneven distribution of research on COVID-19 and human rights in Africa likely also reflects the spread of literature on other health issues. The PRISMA flow diagram (Fig. 2), based on the work of Page and colleagues (2021), shows the selection procedure.

### Study quality assessment

Since we focused on studies using secondary data or conceptual discussions, as were available to June 2021, we adopted a general approach to evaluating their quality, based on the Ways of Evaluating Important and Relevant Data (WEIRD) tool (Lewin et al., 2020). This assesses clarity of paper, completeness of information reported, validity of data sources, and representativeness of analyses, argumentation, ethics, and limitations. Documents were given a rating of no or very minor concerns, minor, moderate, and serious concerns by Diego Chavarro with input from the other authors. We only found concerns for three documents. These included an editorial (Oladimeji et al., 2020), which lacked description of information sources, context, and limitations; an article based on interviews through a WhatsApp group (Dube, 2020), which lacked information on ethical clearance, recruitment procedure, and limitations; and a commentary (Boretti, 2021), where there were weaknesses in aims and in descriptions of sources, programme or intervention, context, and limitations.

We undertook data extraction and analysis simultaneously. Using thematic analysis, we first classified studies into those that addressed vulnerable groups, underlying conditions, human rights, NPIs, and policy proposals. Within these categories we identified specific populations, the underlying conditions co-existing with vulnerability, human rights being protected or violated, policies that were analysed, and policy proposals. These constituted our categories for analysis, allowing us to cluster documents by different themes. These were aggregated and interpreted in relation to our conceptual framework, which changed with new evidence, as explained in the discussion. We also synthesised policy proposals from the literature and developed prompts for policy making. These prompts are not definitive recommendations; rather they provide a guide for implementers to consider within specific policy and regulatory contexts.

### Insights from the literature review

Our analysis is a result of applying a reflexive approach to the literature, described in the previous section. We clustered the documents into overarching avenues of action suggested by scholars, which we classified into: *Recognising and supporting vulnerable groups in policy responses; addressing the underlying conditions of vulnerability;* and *implementing a human rights approach to COVID-19 policies*. They are explained below.

### Recognising and supporting vulnerable groups in policy responses

**V**ulnerable groups are under-represented in government responses to the pandemic, and a few key groups account for the majority in any setting. Women are considered particularly vulnerable, and are seen to be more exposed than men to the virus because of the nature of their work and time spent caring for others (Sekalala, 2020). Women carry the burden of care for their families, and are subject to discrimination as a reflection of patriarchal norms. Women often work without pay, are under-remunerated for other work, and experience greater economic pressures than men, within single parent households and because of the distribution of responsibilities, with lockdowns impeding women from providing for their families and accessing basic health and other services. In Sudan, lockdowns affected women entrepreneurs, most working in the informal economy, as one farmer explained: “COVID-19 is like a death sentence to us vulnerable women who depend on farming to feed our children” (Akech, 2020, p. 595). Govender and colleagues (2020) report that various sexual and reproductive health services in South Africa were suspended because of the prioritisation of COVID-19 treatment, leaving women also with unmet needs for contraceptives, health screening and related exams, and other key services. Gender-based violence is pervasive and reportedly increased during the pandemic (Renzaho, 2020; Sekalala, 2020; Olufadewa et al., 2021); lockdowns and curfews limited women’s capacity to seek help to escape family violence or to report on sexual assault. In West Africa, many women who live in poverty lack sufficient education to be familiar with the law and human rights; even if they report violence, “courts are likely to underestimate the degree of harm faced by women” (Sekalala, 2020, p. 14) and minimise women’s suffering. Few policy measures exist that address women’s vulnerability, and those that exist have been identified as inadequate (Akech, 2020; Amadasun, 2020; Govender et al., 2020; Ntlama and Chitsamatanga, 2020; Renzaho, 2020; Sekalala, 2020; Chiwona-Karltun et al., 2021; Olufadewa et al., 2021).

Children have been identified as especially affected by the pandemic and related NPIs, including, like women, because of increased exposure to violence and illness as a result of containment measures (Akech, 2020; Amadasun, 2020; Dube, 2020; McQuoid-Mason, 2020; Ntlama and Chitsamatanga, 2020; Adebisi et al., 2021; Boretti, 2021). Boretti (2021, p. 1) warns that the mortality of children indirectly related to COVID-19 is expected to be greater in Africa than in other continents. Interruptions to vaccination schedules placed many children at risk of vaccine-preventable diseases (Adebisi et al., 2021). In some countries, such as South Africa, parental visits to children in hospitals were prohibited to limit transmission, leading one author to argue that this “is a clear violation of the child’s right to family care or parental care… likely to undermine the child’s ‘emotional security’” (McQuoid-Mason, 2020). Children’s education was disrupted in African countries and globally, particularly due to the lack of capacity at national and regional levels to provide access to the Internet and support online learning, with immediate and long-term negative consequences (Dube, 2020).

Migrants became increasingly vulnerable because of exclusion from social assistance policies for COVID-19, lack of protection by national laws, a reported rise of xenophobia, and other forms of extreme stigmatisation (Molobe et al., 2020; Mukumbang et al., 2020; Oladimeji et al., 2020; Somse and Eba, 2020; Chiwona-Karltun et al., 2021). Migrants include documented and undocumented foreigners, internally displaced persons in conflict areas, persons who move from rural to urban settings, survivors of human trafficking, undocumented residents, asylum-seekers, and refugees (Molobe et al., 2020; Mukumbang et al., 2020; Odunitan-Wayas et al., 2020). Migrants are disadvantaged by lack of familiarity with their new place of residence, including linguistic and financial constraints, overcrowded living spaces, and precarious employment (Bukuluki et al., 2020; Molobe et al., 2020). This population faces a “lack of consideration … in economic, poverty, and hunger alleviation schemes” (Mukumbang et al., 2020, p. 1). Mukumbang and colleagues argue that “migrants are less willing than nationals to seek testing or care for COVID-19 symptoms” (2020, p. 3), both because of stigmatisation by health and other services due to perceptions that they are vectors of infection (Somse and Eba, 2020) and because of the risk of their identification as residents without official permission, and so fear of deportation or incarceration. However, where policy measures prioritise nationals, even documented persons have problems gaining support, access to food parcels, and essential healthcare (Odunitan-Wayas et al., 2020).

People living in institutions, including prisons (Botes and Thaldar, 2020; Iversen et al., 2020; Muntingh, 2020; Oladimeji et al., 2020), orphanages, and mental health institutions (Iversen et al., 2020), are defined as vulnerable; most of this research has attended to prisons. Many such people live under conditions of extreme deprivation, and pandemic prohibitions on travel and meetings broke the links, inside and outside prison walls and those of other institutions, that previously enabled relatives and prison staff to guarantee basic medicines, food, clothes, and emotional support (Oladimeji et al., 2020; Van Hout, 2020; Yeboah et al., 2020; Chirisa et al., 2021). Living conditions in institutions also heightened the risk of transmission among residents and staff. Further, like health workers in hospitals, prison staff (Van Hout, 2020) and prisoners were likely to infect each other because they shared the same buildings and were in close contact for long periods. Prison staff have low salaries, impacting on their everyday conditions of living, and they were not explicitly supported by social assistance policies for COVID-19.

These four vulnerable groups were most frequently addressed in the literature, and described in greatest detail. In general, the populations disadvantaged by or excluded from COVID-19 policies in Africa to mid 2021 included:

- Women (Akech, 2020; Govender et al., 2020; Ntlama and Chitsamatanga, 2020; Renzaho, 2020; Sekalala, 2020; Chiwona-Karltun et al., 2021; Olufadewa et al., 2021)
- Children (Akech, 2020; Amadasun, 2020; Dube, 2020; McQuoid-Mason, 2020; Ntlama and Chitsamatanga, 2020; Adebisi et al., 2021; Boretti, 2021)
- Youth (Amadasun, 2020; Govender et al., 2020; Renzaho, 2020)
- Migrants, including refugees, asylum-seekers, documented and documented immigrants, persons who migrate from rural to urban environments, internally displaced persons, and trafficked survivors (Bukuluki et al., 2020; Mukumbang et al., 2020; Oladimeji et al., 2020; Somse and Eba, 2020; Chiwona-Karltun et al., 2021)
- Inmates (Botes and Thaldar, 2020; Iversen et al., 2020; Muntingh, 2020; Oladimeji et al., 2020), people in closed settings (Iversen et al., 2020), and prison staff (Van Hout, 2020)
- Persons with medical co-morbidities, including chronic pulmonary disease, cardiovascular disease, cerebrovascular disease, diabetes and compromised immunity (Govender et al., 2020; Iversen et al., 2020; Adebisi et al., 2021; Olufadewa et al., 2021)
- Populations living in extreme poverty, such as those living in informal settlements (Botes and Thaldar, 2020; Odunitan-Wayas et al., 2020; Sehoole, 2020)
- Persons living with disabilities (Botes and Thaldar, 2020; Adebisi et al., 2021), including learners living with severe disabilities (Moodley et al., 2020)
- Professional (Sekalala, 2020; Adebisig et al., 2021; Olufadewa et al., 2021) and non-professional (Sekalala, 2020) healthcare workers. The latter includes birth attendants, community volunteers, traditional healers, and female healthcare providers.
- Homeless populations (Botes and Thaldar, 2020; Oladimeji et al., 2020)
- Sex workers (Iversen et al., 2020; Adebisi et al., 2021)
- People who use drugs, especially those who inject drugs (Iversen et al., 2020; Adebisi et al., 2021)
- Elderly people (Botes and Thaldar, 2020; Olufadewa et al., 2021)
- Sexual minorities, including men who have sex with men and transgender populations (Iversen et al., 2020)
- Individuals in conflict regions (Olufadewa et al., 2021).

The authors of these works call for policy responses, which address the specific needs of people in context. For instance, Adebisi et al. (2021) include social and mental health support for older adults, antenatal care services and medications for pregnant women, access to sexual and reproductive care services for women, COVID-19 information in accessible formats for people living with disabilities, needle exchange and opioid substitution therapy for people who use drugs, access to HIV drugs and care services, access to condoms for sex workers and sexual minorities, and access to health services in prison and other closed settings. Many of the people listed here are marginalised in their own societies, routinely have poor access to services, and are subject to police harassment and risk apprehension. They are rendered invisible or intentionally omitted from programmes to mitigate the effects of NPIs to contain COVID.

While people in these different groups have distinct vulnerabilities and needs, authors also note their intersectionality: that is, people typically belong to more than one group, with compounding disadvantages. Molobe and colleagues (2020) emphasise that women or children, both vulnerable groups, are further disadvantaged when they are immigrants, further still if they are undocumented and therefore anxious to avoid surveillance. Sekalala (2020, p. 4) illustrates the intersectionality in healthcare work, where women’s “race, class, gender, and other axes of oppression overlap.” Odunitan-Wayas and colleagues (2020) point out that during the pandemic, being poor, immigrant, and having a low income all hindered access to food, secure housing, and personal safety, and increased experiences of social injustice. While authors argue the importance of acknowledging diversity and different vulnerabilities, they also highlight overlaps and interactions, and call for specific measures rather than (or in addition to) general measures to limit transmission.

### Addressing the underlying conditions of vulnerability

Different underlying conditions reproduce or aggravate inequalities, leading to new social and structural vulnerabilities. Although we lack evidence for many countries, the economic, health, and social conditions prevalent across the continent heighten the risk of contagion and deepen the vulnerability of poor people. Populations throughout SSA experience high rates of unemployment, limited access to social protection schemes, and unfair employment conditions (Olufadewa et al., 2021); across SSA, informal work contributes “50–80% of gross domestic product, and 60–80% of employment and 90% of new jobs” (p. 5).

Economic conditions deteriorated in African countries during the pandemic (Egger et al., 2021), as illustrated in household surveys conducted during the pandemic in Sierra Leone, Rwanda, Kenya, Ghana, Burkina Faso, and various low- and middle-income countries beyond Africa. While these surveys were not statistically representative of national populations, the authors identified drops in income and employment, reduced access to markets, delayed healthcare access, missed or reduced meals, and increasing dependence on NGO or government support (Egger et al., 2021).

Reports that lockdown measures threatened livelihoods and exacerbated inequalities are widespread. Chiwona-Karltun and colleagues (2021), using a gender lens, analysed data from 12 Sub-Saharan countries—Benin, the Democratic Republic of Congo, Ghana, Cote d’Ivoire, Kenya, Mozambique, Nigeria, Rwanda, South Africa, Tanzania, Uganda, and Zambia—to understand people’s concerns during lockdowns, and noted both men and women were concerned that lockdowns would impact economic and food security. Odunitan-Wayas, Alaba and Lambert likewise observed that “the triple burden of food insecurity, poverty and malnutrition compounded with social injustice and income inequality (was) inevitable for the urban poor African immigrants in South Africa” (Odunitan-Wayas et al., 2020, p. 151). Authors call for policy responses to directly address these interrelated underlying conditions, and argue that measures such as cash payments and financial relief are ineffective, insufficient, or palliative (Renzaho, 2020; Adebisi et al., 2021; Boretti, 2021).

Other studies confirm that economic conditions arising from COVID-19 policies had direct negative affects. Foreign-born migrants, asylum-seekers, and undocumented migrants (Mukumbang et al., 2020) needed to find alternative means of income when movement was restricted, often increasing their risk of infection and incarceration for breaking isolation and quarantine measures (Moodley et al., 2020). The majority of elderly people in SSA do not have access to pensions, and most rely on sources of income restricted by NPIs (Renzaho, 2020). In countries where extreme poverty is widespread, such as Malawi, people faced increasing prices of goods, and lacked commercial and employment opportunities, and children lost access to meals that were provided at schools when these were closed (Nkhata and Mwenifumbo, 2020).

Vulnerable groups are particularly affected by poor health systems and limited access to healthcare. Renzaho (2020, p. 2) emphasises that now as decades ago, communicable, maternal, neonatal, and nutritional (CMNN) diseases, including HIV/AIDS, are prevalent; COVID-19 deflected attention from these conditions. Renzaho (2020, p. 4) also notes that still “the majority of women giving birth do not have access to maternity cash benefits,” leading to insufficient protection of life even when benefits are available in other social welfare and protection programs. The capacity of states to address maternal and child health, infections and non-communicable diseases, are especially problematic where civil unrest is endemic, where countries have poor infrastructure and inadequate medical facilities, materials and professionals (Sekalala, 2020, p. 2). People living in the everyday circumstances of violence and poverty are likely to suffer from multiple physical and mental health conditions (Mukumbang et al., 2020).

War and conflict aggravate vulnerability, precipitating flight (Akech, 2020). The experiences of the pandemic on people who live in temporary, contained living conditions, including refugee camps, have yet to be described. On migration and resettlement, structural vulnerabilities compromise their health and well-being, and they face xenophobia and structural barriers to care and support. Most asylum-seekers, refugees, and undocumented immigrants come from places where communicable diseases are endemic, and these continue in new settings.

Health workers are embedded in a hierarchy, within which well-paid workers delivering international humanitarian aid, with formal contracts and health support systems, are at the top, while national healthcare professionals have weaker employment conditions and do not enjoy the possibility to be taken to a first-tier hospital if required (Sekalala, 2020). Sekalala (2020) argues that non-professional caregivers are at the bottom of this hierarchy, with women the least recognised and rewarded, struggling to get access even to basic personal protective equipment. Throughout SSA, as discussed above, women are subordinate and expected to perform roles that put them at high risk of contagion, discrimination, and economic burden.

Sexual minorities are widely excluded by dominant social values, and these values were reproduced in COVID-19 policies (Adebisi et al., 2021). Men who have sex with men, transgender people, and sex workers were excluded from social protection mechanisms in most countries, were already often subject to regular harassment, and could not access appropriate health support (Adebisi et al., 2021). Prisoners and people in other closed settings also face exclusion, stigma and discrimination, and are deprived of basic healthcare and hygiene (Kassa and Grace, 2020; Muntingh, 2020; Mekonnen et al., 2021). During the pandemic, the health conditions of inmates and prison staff were threatened by “old physical infrastructure, insufficient sanitation, ventilation and hygiene, (and) severe congestion” (Van Hout, 2020, p. 128).

### Implementing a human rights approach to COVID-19 policies

Certain rights were restricted to curb the spread of COVID-19 during the pandemic. Governments across Africa declared states of emergency and introduced disaster laws, and these variously protected or infringed human rights, leading scholars to call for a reasonable and proportionate response, using the least intrusive interventions available (Akech, 2020; Nkhata and Mwenifumbo, 2020; Nkuubi, 2020).

Distinct from the personal issues we describe above, states of emergency and disaster laws threatened the right to periodic elections. Ethiopia and Chad postponed legislative and parliamentary elections, and other countries contemplated the same (Renzaho, 2020). While postponing elections may have been justified by the severity of the pandemic and problems with mass gatherings, this decision could be abused by some to retain or gain power (Nkuubi, 2020; Renzaho, 2020).

There was expressed concern in the literature about the use of special powers by governments, particularly militarisation to contain transmission (African Union, 2020). Amadasun (2020, p. 1) refers to “widely reported cases of violence against citizens by security forces who were deployed to enforce curfews and lockdowns” in Kenya, South Africa, and Nigeria. Manderson and Levine (2021) describe soldiers strong-arming homeless residents and people living in informal settings in South Africa. Mukumbang et al. (2020, p. 3) report that organisations for migrants have denounced “the arrest and detention of foreign-born migrants, their placement in, and subsequent repatriation from camps and shelters.” Others living in precarious conditions, such as persons who inject drugs and sex workers, were targeted by the police during the pandemic more so than usual (Iversen et al., 2020).

The deployment of armed soldiers to enforce the rules decreed by public health laws is not the only instance of militarisation. The pandemic was communicated as a war against a common enemy, and this narrative was used to justify the restriction of human rights. Nkuubi (2020), for instance, shows that in Uganda the rhetoric of war was common in government media campaigns, and this was literalised by the appointment of military staff to directive positions in most civilian institutions that dealt with the pandemic, including hospitals. In South Africa, militarisation built upon repressive structures and technologies, including those derived from apartheid (Manderson and Levine, 2020, 2021). Addressing the pandemic as a war reinforced dominant patriarchal hierarchies, leading to the increased abuse of women (Sekalala, 2020). In addition, the criminalisation of people who ignored curfews, lockdowns, and face mask wearing increased atmospheres of fear and anxiety in vulnerable populations (Akech, 2020; Chiwona-Karltun et al., 2021).

In enforcing lockdowns, governments limited human rights and failed to guarantee them. Freedom of opinion and expression was constrained as political opponents and protesters in some instances were detained and prosecuted (Iversen et al., 2020; Okolie-Osemene, 2021; Olufadewa et al., 2021). The right to education was limited when schools were closed for an undetermined period and school systems were unable to support learners with disabilities or, as was common, with poor access to internet (Dube, 2020; Kamga, 2020; Beckmann and Reyneke, 2021). The right to work was affected because of restrictions to movement, whether or not justified by the severity of the pandemic (Fombad, 2020; Molobe et al., 2020; Nkhata and Mwenifumbo 2020; Odunitan-Wayas et al., 2020). Policies restricted all sectors of the economy, and governments often failed (and lacked fiscal and administrative capacity) to implement measures to ensure the right to food and shelter for those who were most vulnerable (Nkhata and Mwenifumbo, 2020; Odunitan-Wayas et al., 2020; Chiwona-Karltun et al., 2021). Freedom from discrimination was violated where social assistance policies ignored the needs of particular sectors of the population, and as described above, some of these categories are criminalised (Molobe et al., 2020; Mukumbang et al., 2020; Muntingh, 2020; Oladimeji et al., 2020; Van Hout, 2020). Further, the right to health was compromised where state provision of medicines and services were interrupted and private health institutions were not affordable (Govender et al., 2020; Iversen et al., 2020; Oladimeji et al., 2020; Sehoole, 2020; Sekalala, 2020; Somse and Eba, 2020; Adebisi et al., 2021).

The boundaries between reasonable and unreasonable responses by governments can be difficult to establish, and national courts are central in interpreting the law and limiting the exercise of power. Nkhata and Mwenifumbo (2020, p. 525) report that the National Court of Malawi reviewed different measures decreed by the government, and concluded that, in light of the severity of the pandemic at that moment, the Coronavirus Rules “exceeded the authority provided by the parent Act, namely, the Public Health Act;” this prevented a lockdown. Conversely, in Kenya, the Court ruled that while no data were available on the effectiveness of lockdown measures, a curfew was constitutional on the precautionary principle (Kabira and Kibugi, 2020). In other countries such as Eswatini, Lesotho, and South Africa, national courts played a decisive role (Fombad, 2020; Shale, 2020; Shongwe, 2020; van Staden, 2020), only partially endorsing government actions which violated constitutional rights (van Staden, 2020).

Government policy measures influence the behaviour of civilians, leading to further threats to human rights. As noted above, Amadasun (2020, p. 1) documents that “gender-based violence have intensified in countries where promulgation of shutdown or stay-at-home orders have been implemented,” and Akech (2020) reported that sexual violence against girls in South Sudan increased during lockdowns. Mukumbang et al. (2020) described an increase in violence against foreigners in South Africa associated with COVID-19 stigma; this erupted into riots in mid 2021. Iversen and colleagues (2020) foreshadowed that sex workers and drug users would be even more exploited by clients and drug dealers. Researchers have identified an increase in violence indirectly related to stringent policy measures, while also revealing increasing inequalities on the continent.

Several policy recommendations emerge to ensure that policy responses to pandemics protect the human rights of vulnerable populations, although how these might be translated and implemented will vary in different countries. They include:

- *Achieving a balance between the containment of infection and protection of human rights*. Policy responses aimed at containing COVID-19, and preventing the spread of infection in future pandemics, need to balance measures to avoid harm to vulnerable people (Govender et al., 2020; McQuoid-Mason, 2020; Moodley et al., 2020; Muntingh, 2020; Sehoole, 2020; Sekalala, 2020; Shongwe, 2020; Somse and Eba, 2020; Chiwona-Karltun et al., 2021; Olufadewa et al., 2021). Laws decreed for emergencies and disasters often violate international human rights conventions and national constitutions, and authors call for governments to abide by the internationally agreed standards such as the Siracusa principles to produce responses that are “legally and ethically justifiable under particular circumstances, [and offer] a fair measure of compassion, restraint and respect for human rights” (Moodley et al., 2020, p. 2). Recommendations include consulting with relevant institutions of the state before declaring states of emergency and disaster (Akech, 2020), allowing and promoting internal and international oversight of the policy process (Muntingh, 2020; Chiwona-Karltun et al., 2021), and ensuring transparency and accountability (Shongwe, 2020).
- *Implementing population-specific rather than uniform responses*. There is a clear need to identity populations not well represented in or excluded from general social and economic policies and programs. Curfews and lockdowns, for example, disproportionately affect marginalised groups by limiting their access to food, health services and medicines, lowering their incomes, increasing their exposure to xenophobia and exploitation, and exposing them to other forms of violence that violate their human rights (Govender et al., 2020; Iversen et al., 2020; Molobe et al., 2020; Moodley et al., 2020; Muntingh, 2020; Oladimeji et al., 2020; Parker et al., 2020; Renzaho, 2020; Adebisi et al., 2021; Olufadewa et al., 2021).
- *Treating the causes instead of the symptoms*. Including vulnerable groups and responding to the conditions of vulnerability in policies implies that the “social policy response must address root causes that elevate the vulnerability of people to abuses” (Amadasun, 2020, p. 2). These include weak health systems, increasing privatisation of health, exclusion of people living in informal settings or working in the informal economy, and income and other inequalities (Akech, 2020; Kamga, 2020; Muntingh, 2020; Oladimeji et al., 2020; Sehoole, 2020; Sekalala, 2020; Somse and Eba, 2020; Van Hout, 2020; Beckmann and Reyneke, 2021; Chiwona-Karltun et al., 2021; Olufadewa et al., 2021) To identify appropriate action and protection, we need better data on vulnerable groups and their disproportionate experiences of poverty, discrimination and stigma.
- *Planning rather than reacting*. In light of the real sense of urgency during the pandemic, government responses were often reactive, enforced by police, the military and at times private security forces, with harsh action against people for minor infringements (Amadasun, 2020; Iversen et al., 2020; Manderson and Levine, 2020; Mukumbang et al., 2020; Nkuubi, 2020; Parker et al., 2020; Okolie-Osemene, 2021; Olufadewa et al., 2021). There is strong documentation of violence against and distress among citizens as NPIs were implemented and enforced (Olaseni et al., 2020), leading to contestation in national courts (Nkhata and Mwenifumbo, 2020; Shale, 2020, Shongwe, 2020). Authors recommend enhancing social protection systems to be responsive to crises (Renzaho, 2020), updating legislation that derives from and still reflects the colonial past (Muntingh, 2020), retraining law enforcement officials and civil servants in human rights (Amadasun, 2020), designing laws to include social protection measures for people regardless of national identity or any type of official registry (Mukumbang, Ambe et al., 2020), and enhancing capacity to anticipate the social, economic, and environmental consequences of interventions (Ntlama and Chitsamatanga, 2020, Chiwona-Karltun et al., 2021).
- *Co-constructing rather than instructing*. In planning exercises, strategies are needed to minimise the unintended effects by designing interventions in collaboration with different groups and stakeholders such as NGOs (Akech, 2020; Govender et al., 2020, Iversen et al., 2020; Odunitan-Wayas et al., 2020; Olufadewa et al., 2021). This lesson was clearly learned during the Ebola and HIV crises. A similar approach could ensure that “the COVID-19 response, or ‘cure’, is not worse than the disease itself” (Iversen et al., 2020, p. 2).

## Discussion and concluding remarks

In reviewing the literature, we offered a conceptual framework to analyse public policy responses to COVID-19, which included structural vulnerability in analysis, and we examined the impacts of NPIs on the health and human rights of vulnerable populations. From this we considered the implications of this review for policy making.

As explained above, structural vulnerability refers both to the social structures and institutionalised conditions that position some people as more vulnerable than others. People who lack the right to healthcare or social support, for example, are structurally vulnerable because of the precondition of citizenship or legal resident status to access support, and they necessarily organise their lives to avoid being apprehended and deported. In this respect they are structurally vulnerable. Their vulnerability may be compounded by gender, ethnicity, or occupation. As we have argued, social status, citizenship, economic status and health are interrelated, and those who were already vulnerable and disadvantaged prior to the pandemic were especially vulnerable under COVID-19 regulations.

Our initial framework (Fig. 1, above) considered structural vulnerability as resulting from and affected by the interrelationships between human rights, health, and socio-economic underlying conditions. Based on this, we examined the literature to identify different vulnerable groups affected by COVID-19 regulations, their underlying conditions, and the impacts of regulations on their human rights. As described in the literature, we found (1) a large yet incomplete list of vulnerable groups, whose members are excluded from or not adequately represented in policy responses to COVID-19; (2) the precarious socio-economic conditions of these populations are not adequately addressed by dominant policy responses; and (3) the human rights of these populations are threatened or violated. Dominant policy responses to COVID-19 recognised people who fell within the state’s ambit of citizenship; only partial support was offered to those who do not, and whose relationship with the state was ambiguous or conditional. This reinforced structural vulnerability, structural violence, the increased risk of people who are situationally vulnerable, and the risks for people confined to institutions.

**Fig. 3:**
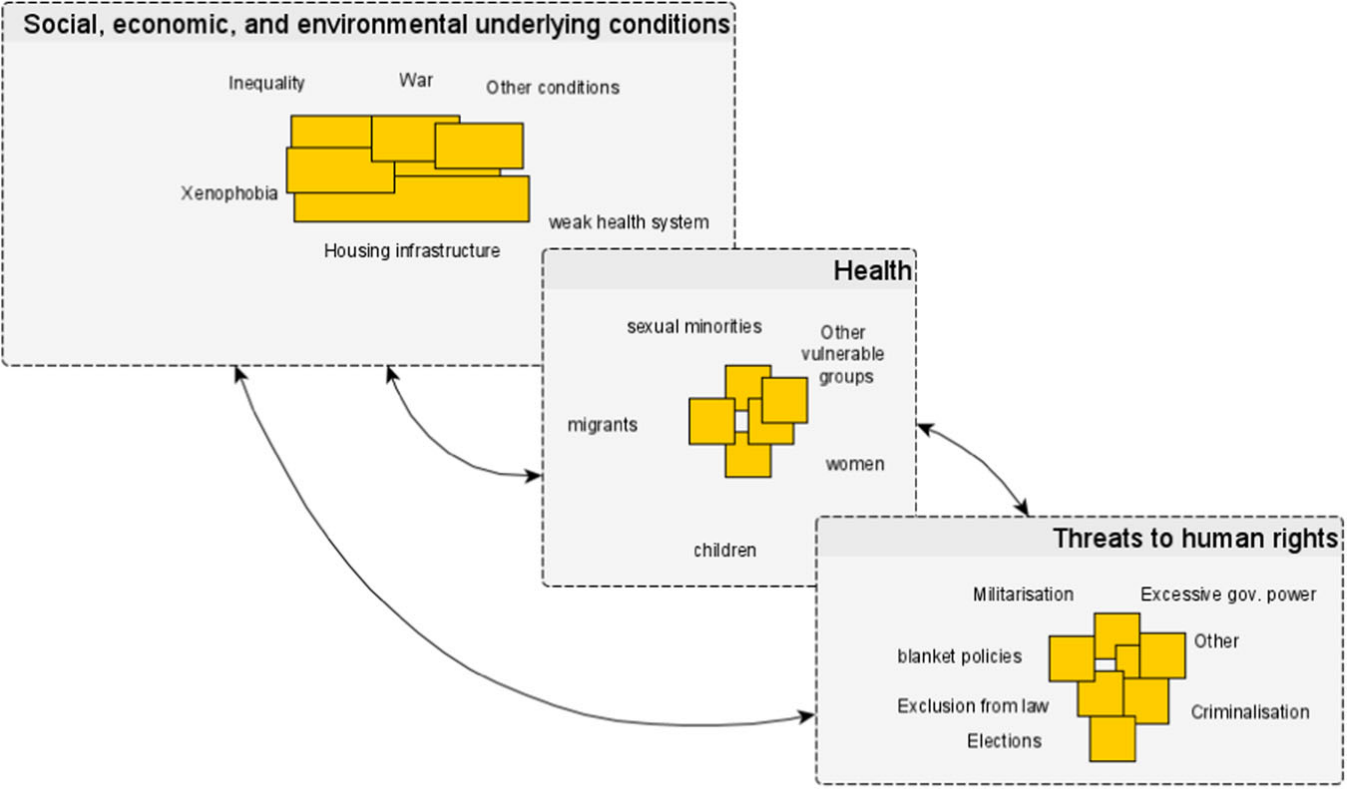
Refined conceptual framework. This includes the concept of “recursive cascades” to explain the relationship between social, economic, and environmental underlying conditions; health; and threats to human rights.

The effects of intersectionality on vulnerable groups are clear from evidence from various countries on the continent. Intersectionality emphasises that structural conditions, such as gender, race, and class, interact with each other. The interactions between health, human rights, and underlying conditions can produce “recursive cascades,” a metaphor used “to capture the often inevitable trajectory of increasing ill health and growing impoverishment” (Manderson and Warren, 2016, p. 479). COVID-19 restrictions and vulnerability to disease increase other vulnerabilities, with compounding effects. Take as an example a young woman living in a society, in which inequality and insecure work without social protection, prevail under any circumstance. She is more prone during COVID-19 lockdowns to violation of her human rights, including physical and sexual violence and worsened living conditions; in turn, the violation of her human rights makes her especially vulnerable to contracting the virus and not receiving timely access to care. At each point of her experience, the circumstances and conditions of vulnerability interact with and amplify other vulnerabilities. To capture this, drawing on the concept of recursive cascades, we suggest a modification to our framework (Fig. 3), which attempts to contribute to the planning and analysis of policy responses to COVID-19.

This framework can be used to consider policy interventions in relation to their interactions and causes, and to anticipate the potential effects of interventions in the context of pandemic preparedness. In this framework identified factors can exist concurrently, with overlaps within and between categories. This identifies the need to design policies that take account of multiple societal dimensions of health, rather than only its biomedical components.

In the literature, scholars call for COVID-19 policies that

- achieve a balance between COVID-19 spread and the protection of human rights;
- implement population-specific responses to supplement uniform public health responses;
- treat the causes instead of the symptoms;
- plan instead of react; and
- co-construct with instead of instructing people, especially vulnerable populations.

We present these recommendations as instruments to be considered when designing new policies in relation to pandemic preparedness, so to incorporate a human rights perspective.

## Limitations

Our familiarity and interest in this subject influenced our literature search and conclusions. Specifically, we looked for papers addressing impacts on the human rights and health of vulnerable populations, focusing our attention on a debated subject that goes beyond biomedical understandings of health. Because of our integral understanding of health, we have constructed a conceptual framework (above) that may not fit with dominant medicalised approaches to health, although it is in accordance with a growing understanding of health as socially mediated (WHO Commission on Social Determinants of Health, 2008). We are aware that identifying human rights is likely to denote violations rather than address the protection of human rights. This may have biased our results towards the negative impacts of policies. Our aim, however, was to contribute to a body of literature that considers the importance of understanding negative impacts to inform policy to achieve human well-being. We did not intend to include all literature on the subject, but rather sought to bring a conceptual understanding of health policy in the broader framework of human rights and structural vulnerability. Our findings provide a basis to infer other possible impacts, underlying conditions, vulnerabilities and vulnerable populations, potential interventions and policy recommendations. We identified some characteristics of the research on human rights and its relationship with COVID-19 policies. The first is that most documents are commentaries or editorial pieces, the majority from the perspective of the law. Few documents were based on primary data, such as derived from interviews, surveys, or ethnography. This is likely due to the recency of the pandemic, limiting the availability of data for complex issues such as the relationship between policy, human rights, and COVID-19. A second characteristic of the literature is that most of it focused on South Africa, leaving other Sub-Saharan African countries with few or no studies, thus impeding a more encompassing understanding of human rights during the pandemic. This may reflect research capacity concentration, which is a long-standing issue in Africa. These two characteristics show the need to produce primary data on the relationship between human rights, policies, and health; to support research that uses this data; and to expand the geographic focus of research and the research capacities on the continent.

## Data Availability

This article is based on the analysis of publications available to June 2021, and we did not generate new datasets. Search strategies are provided in the Appendices in Chavarro et al. (2021).
